# Regarding the Necessity of Functional Assessment Including Motor Control Assessment of Post-Mastectomy Patients Qualified for Latissimus Dorsi Breast Reconstruction Procedure—Pilot Study

**DOI:** 10.3390/ijerph17082845

**Published:** 2020-04-21

**Authors:** Rita Hansdorfer-Korzon, Damian Wnuk, Jakub Ławnicki, Maciej Śliwiński, Agnieszka Gruszecka

**Affiliations:** 1 Department of Physiotherapy, Faculty of Health Sciences, Medical University of Gdańsk, Dębinki 7, 80-211 Gdańsk, Poland; rita.hansdorfer-korzon@gumed.edu.pl (R.H.-K.); damian.wnuk@gumed.edu.pl (D.W.); jakub.lawnicki@gumed.edu.pl (J.Ł.); 2 Department of Radiology Informatics and Statistics, Faculty of Health Sciences, Medical University of Gdańsk, Tuwima 15, 80-210 Gdańsk, Poland; agruszecka@gumed.edu.pl

**Keywords:** breast cancer, latissimus dorsi breast reconstruction, shoulder function, functional assessment, motor control assessment

## Abstract

The purpose of the paper is a functional assessment of post-mastectomy patients who underwent latissimus dorsi breast reconstruction (LDBR), and of healthy women, through an analysis of selected muscle function parameters, including motor control assessment. Twenty participants were included in the study (ten LDBR-procedure individuals and ten healthy controls). The research consisted of a DASH (The Disabilities of the Arm, Shoulder and Hand) questionnaire assessment, shoulder area static assessment, shoulder mobility assessment, latissimus dorsi flexibility assessment and shoulder motor control assessment. LDBR-procedure individuals—when compared to healthy controls—exhibited a decrease in physical aspects of quality of life, shoulder area postural alterations, limitations in shoulder mobility and decrease in shoulder motor control. LDBR procedure may have an influence on limiting shoulder active mobility, as well as on decrease of shoulder motor and postural control. Standard functional assessment diversified on motor control assessment of post-mastectomy patients qualified for the LDBR procedure seems to be necessary.

## 1. Introduction

Breast cancer is the most prominent oncological issue in the highly developed countries and is further becoming one in the developing countries. The prognosis for the years 2010–2025 in Poland points to an increase in the number of patients of all age groups [1]. Surgical breast removal caused by cancer in a large number of patients can be followed by reconstruction provided there are no contraindications [2]. Moreover, a European Parliament resolution on breast cancer in the European Union (2002/2279[INI]) states that: “whenever possible, the breast should be reconstructed using autologous tissue and the procedure should be conducted as soon as possible” [3]. Available options include various techniques and if at all possible, the reconstruction should be conducted simultaneously with the mastectomy to limit the change of body perception or in a delayed mode a minimum six months after the amputation and after ruling out any active cancer process. The reconstruction procedure may be conducted using autologous tissue in the form of musculocutaneous flaps either pedunculated on microsurgical attachments or, which is less strenuous for the body, using tissue spreaders and breast prosthetics or Becker prosthetics [4]. The procedure should ensure the best possible not only aesthetical but also functional result. In the case of qualifying a patient for delayed reconstruction, it should also be remembered that due to the breast amputation and consequently any pain and limited mobility of the upper limb, body statics become disrupted [5,6].

The latissimus dorsi is primarily described as a muscle responsible for shoulder movements. However, due to its attachments to thoracic and lumbar vertebrae, sacrum and ilium, it also plays a role in trunk mechanics and upper limbs movements may additionally influence its functioning [7]. It is assumed that the latissimus dorsi is responsible for adduction, extension and internal rotation of the shoulder joint [8]. This muscle is probably an important part of force transmission among the upper limb, the trunk and the lower limb due to its myofascial attachment with the gluteus maximus [9].

Due to the high vascularity and surgical accessibility, the latissimus dorsi finds a variety of uses in various kinds of reconstruction procedures [10,11]. However, utilizing this surgical solution may result in certain functional limitations [10,12,13,14], such as muscle strength reduction, pain and negative influence on the patient’s life quality [10,15]. The procedure may also cause a permanent change in muscle length, which may result in restricted upper limb mobility [16]. The transposition of a fragment of the latissimus dorsi causes function change of the muscle and consequently changes to shoulder biomechanics [17].

Due to the possible occurrence of functional limitations as described above as well as functional changes described by numerous authors after the mastectomy itself, the implementation of a functional assessment of patients after a delayed procedure using latissimus dorsi flap (LDF) as a part of comprehensive and interdisciplinary treatment seems necessary. On the other hand, none of the studies had included motor control assessment as a part of examination.

The purpose of the paper is a functional assessment of post-mastectomy patients who underwent latissimus dorsi breast reconstruction (LDBR) and of healthy women, through an analysis of selected muscle function parameters, including motor control assessment. This study follows the principles of the Declaration of Helsinki.

## 2. Materials and Methods

The tests concerned 20 participants, 10 of whom were the research group (group A), and 10 of whom were a control group (group B). Group A (mean age 47.5; from 34 to 63) contained patients who underwent surgery due to breast cancer and an autologous delayed latissimus dorsi breast reconstruction procedure. Average time from mastectomy to LDBR was 14.3 months (from 2 to 36). The tests were conducted once, within a year of the reconstruction procedure. Group B (mean age 40.3; from 35 to 51) consisted of healthy and professionally active women who did not undergo any surgery of upper limbs, trunk or abdomen, experienced no trauma of upper limbs or trunk and were not diagnosed with cancer.

The research consisted of two parts: an assessment with a DASH (The Disabilities of the Arm, Shoulder and Hand) questionnaire (http://www.dash.iwh.on.ca/) and a physical examination. The physical examination consisted of shoulder area static assessment, shoulder mobility assessment, latissimus dorsi flexibility assessment and shoulder motor control assessment. A DASH questionnaire was used in order to subjectively assess the degree of upper limb disability.

Static assessment consisted of positional evaluation of both scapulae in a relaxed standing position. Distances between osseous landmarks of the scapula (lower and upper angle) and spinous processes line were analyzed.

Shoulder mobility assessment consisted of goniometric measurement of flexion, extension, abduction and external rotation in active movements.

In order to assess latissimus dorsi flexibility, the examined patient bent both upper limbs (with extended elbow joints) in the movement’s full possible range. The test was conducted in a sitting position on the floor, with maximally bent hips and knees (knees brought to the chest) and back leaning on a wall. At the end of the movement range, the value of the angle between the shoulder and the wall was recorded.

Motor control assessment consisted of shoulder movement dissociation tests described by Comerford and Mottram [18]. During the tests, the ability to actively control and stabilize the scapula while moving the shoulder joint (flexion, extension, abduction and external rotation) was assessed. The tests were conducted individually for each limb. Markers facilitating movement observation were placed on selected osseous landmarks—lower and upper angles of the scapula and acromion. Each examined movement was recorded with a video camera and then analyzed by the researchers. It was assumed that scapula should remain stability during initiation of shoulder movement and its first phase—the angle of shoulder movement in which scapular motion occurred was recorded (the lower angle the worse neuromuscular control of the movement).

For the statistical assessment in group A, the quotient of the operated side and non-operated side was determined, while the quotient of the left side and the right side was used in group B. The Shapiro–Wilk test was used for normality assessment and the Levene’s test was used to assess the equality of variances. The *t*-test for independent variables was subsequently used to analyze variables meeting the requirements of the parametric test, while the non-parametric Mann–Whitney U test was used to analyze variables not meeting these requirements. The assumed statistical significance level was *p* ≤ 0.05. The Bonferroni correction was used to counteract the problem of multiple comparisons. The value of statistical significance after correction will decrease to the value of approximately 0.004. However, the probability of majority of individual hypothesis is below 0.004, so the significance of the desired overall alpha level remains at 0.05. The G*Power 3.1.9.2 software (Heinrich Heine Universitat, Dusseldorf, Germany) was used to calculate tests power (*p*). In this calculation post hoc analysis was conducted.

## 3. Results

### 3.1. Assessment with DASH Questionnaire

In group A, the average DASH (The Disabilities of the Arm, Shoulder and Hand) questionnaire result was 60.7 points (from between 30 to 98 points). In group B, the average result was 32.6 points (from between 30 to only 42 points).

Obtained results suggest that the group of patients assessed the degree of upper limb disability as higher than the control group. The results were statistically significant (Table 1).

### 3.2. Scapular Position Static Assessment

In group A, the distance between the scapular lower angle and the spine line was greater on the operated side for 90% of the patients. In the case of asymmetry, the average value in this group was as much as 1.2 cm (from 0.5 to 2.5 cm). In group B, differences in the distance of scapular lower angle between the left side and the right side were noted for 70% of respondents. Nonetheless, the average value in this group was 0.45 cm (from 0.3 to 1.2 cm). Obtained results point to a higher level of lower angle distance asymmetry in the examined group (operated side in relation to non-operated side) compared to the control group. The results were statistically significant (Table 2). In group A, the distance between the scapular upper angle and the spine line was longer on the operated side for 70% of the patients. In the case of asymmetry, the highest value in this group was as much as 3.0 cm, while the average value was 1.0 cm. In group B, asymmetry in upper angle distance from the spine line was observed for 80% of respondents. However, it should be noted that the highest asymmetry value was only 1.0 cm, and the average value was a mere 0.45 cm. Obtained results point to a higher level of upper angle distance asymmetry in the examined group (operated side in relation to non-operated side) compared to the control group. The results were statistically significant (Table 2).

### 3.3. Shoulder Active Mobility Assessment

**Flexion:** In group A, the average range of motion (ROM) value during shoulder flexion on the operated side was 148.3° (from between 120° to 175°). The ROM value on the non-operated side was 164° (from between 153° to 175°). The ROM was smaller on the operated side for 80% of patients. In the case of asymmetry, the average value was 15.7° (from between 4° to 33°). In group B, asymmetry between the left side and the right-side during flexion was noted for 100% of respondents, however, the average asymmetry value was only 3.8° (from between 1° to 7°). Average flexion range value for both limbs was 166.4° (from between 150° to 174°). Obtained results point to a higher level of flexion ROM asymmetry in the examined group (operated side in relation to non-operated side) compared to the control group. The results were statistically significant (Table 3).

**Abduction:** In group A, the average movement range value during shoulder abduction ROM on the operated side was 136.8° (from between 80° to 178°). The average abduction ROM on the non-operated side was 172.1° (from 160° to 180°). The ROM was smaller on the operated side for 100% of patients. The average asymmetry value was as much as 35.3° (from 2° to 91°). In group B, asymmetry between the left-side and the right-side during abduction was noted for 100% of respondents, however; the average asymmetry value was only 4.0° (from between 1° to 14°). Average abduction ROM value for both limbs was 168.1° (from between 155° to 178°). Obtained results point to a higher level of abduction ROM asymmetry in group A (operated side in relation to non-operated side) compared to group B. The results were statistically significant (Table 3).

**Extension:** In group A, the average extension ROM on the operated side was 15.9° (from between 7° to 28°). The average ROM value on the non-operated side was 19.2° (from between 10° to 33°). The ROM was smaller on the operated side for 60% of patients. The average asymmetry value was 3.3° (from between 4° to 8°). In group B, asymmetry between the left side and the right- side during shoulder extension was noted for 80% of respondents, while the average asymmetry value was only 1.8° (from between 1° to 5°). Average ROM value for both limbs was 21.3° (from between 17° to 28°). Obtained results point to a higher level of extension ROM asymmetry in the examined group (operated side in relation to non-operated side) compared to the control group. The results were statistically significant (Table 3).

**External rotation:** In group A, the average external rotation ROM value on the operated side was 38° (from between 5° to as much as 75°). The average ROM value on the non-operated side was 52.1° (from 20° to75°). The ROM was smaller on the operated side for as much as 90% of patients. The average asymmetry value was 14.1° (from between 1° to 29°). In group B, asymmetry between the left side and the right side in external rotation ROM was noted for 100% of respondents, however; the average asymmetry value was significantly lower and equaled 3.9° (from between 2° to 11°). Average external rotation range value for both limbs was 51.75° (from between 25° to 61°). Obtained results point to a higher level of external rotation ROM range asymmetry in the examined group (operated side in relation to non-operated side) compared to the control group. The results were statistically significant (Table 3).

### 3.4. Latissimus Dorsi Flexibility Assessment

In group A, the average shoulder flexion angle value on the operated side was 146.7° (from between 103° to 168°), with the average on the non-operated side being 158.27° (from between 136° to 173°). The angle value was lower on the operated side for 90% of patients. In the case of asymmetry, the average difference was 13.17° (from between 1° to 33°). In group B, the average shoulder flexion angle value on both sides was 164.67° (from between 145° to 174°). Asymmetry between limbs was observed in 100% of women, however; it should again be noted that average difference was only 4.0° (from between 1° to 10°). Obtained results point to a higher level of asymmetry in the latissimus dorsi flexibility test in the examined group (operated side in relation to non-operated side) compared to the control group. The results were statistically significant (Table 4).

### 3.5. Motor Control Assessment

**Flexion:** In group A, the average value of the angle at which scapular control loss occurred during shoulder flexion on the operated side was 65.5° (from between 20° to as much as 160°). Average value of the angle on the non-operated side was 108.9° (from between 30° to 175°). The control loss angle value was lower on the operated side for 90% of patients. In the case of asymmetry, the average value was 43.4° (from between 10° to 99°). In group B, asymmetry between the left side and the right-side during shoulder flexion was noted for 50% of respondents. Average asymmetry value was 16.6° (from between 3° to as much as 115°). Average movement control loss angle value for both limbs was 131.8° (from between 50° to as much as 173°). Obtained results also point to a higher level of asymmetry of scapular position control loss angle during flexion in the examined group (operated side in relation to non-operated side), compared to the control group. The results were statistically significant (Table 5).

**Abduction:** In group A, the average value of the angle at which the scapular control loss occurred during shoulder abduction on the operated side was 75.8° (from between 20° to as much as 155°). Average value of the angle on the non-operated side was 151.5° (from between 70° to 180°). The control loss angle value was lower on the operated side for 100% of patients. In the case of asymmetry, the average value was 75.7° (from between 20° to 139°). In group B, asymmetry between the left-side and the right-side during abduction was noted for 100% of respondents. Average asymmetry value was only 5.5° (from between 1° to 14°). Average movement control loss angle value for both limbs was also lower and equaled 138.75° (from between 50° to 178°). Obtained results also point to a higher level of asymmetry of scapular control loss angle during abduction in the examined group (operated side in relation to non-operated side) compared to the control group. The results were statistically significant (Table 5).

**External rotation:** In group A, the average value of the angle at which the scapular control loss occurred during shoulder external rotation on the operated side was 28.6° (from 0° to 50°). Average value of the angle on the non-operated side was 50.2° (from between 0° to as much as 75°). The control loss angle value was lower on the operated side for 90% of patients. In the case of asymmetry, the average angle value was 21.6° (from between 1° to 57°). In group B, asymmetry between the left side and the right-side during shoulder external rotation was noted for 40% of women. The average asymmetry value was only 2.7° (from between 2° to 20°). Average movement control loss angle value for both limbs was 44.45° (from between 10° to 60°). Obtained results also point to a higher level of asymmetry of scapular control loss angle during external rotation in the examined group (operated side in relation to non-operated side) in comparison to the control group. The results were also statistically significant (Table 5).

**Extension:** In group A, the average value of the angle at which the scapular control loss occurred during shoulder extension on the operated side was 12.2° (from between 0° to 20°). Average value of the angle on the non-operated side was 17.2° (from between 10° to 23°). The control loss angle value was lower on the operated side for 70% of patients. In the case of asymmetry, the average value was 5° (from between 4° to 10°). In group B, asymmetry between the left side and the right side in the shoulder extension was noted for 60% of respondents. Average asymmetry value was only 1.4° (from between 1° to as little as 5°). Average movement control loss angle value for both limbs was 19.2° (from between 5° to 27°). Obtained results point to a higher level of asymmetry of scapular control loss angle during extension in the examined group (operated side in relation to non-operated side) compared to the control group. The results were statistically significant (Table 5).

## 4. Discussion

Breast cancer is a disease from which millions of women suffer, many of them at a relatively young age. Post-mastectomy reconstruction alleviates the emotional and physical toll of this devastating disease. Arora et al. showed in their research conducted during 2001, that a group of women who underwent the reconstruction emerged in far better emotional condition than women who underwent mastectomy without reconstruction surgery [19]. Ueda et al. also evaluated post-operative life quality; patients who underwent mastectomy without accompanying reconstruction surgery assessed their own appearance as significantly worse than before the treatment [20]. Other data gathered by Polish researchers shows that 80% of women who underwent reconstruction believed the surgery increased their attractiveness [21]. Recommendations from the National Institute for Health and Clinical Excellence (NICE), “Early and advanced breast cancer: diagnosis and treatment”, from February 2009 contain the following opinion: “Discuss immediate breast reconstruction with all patients who are being advised to have a mastectomy and offer it except where significant comorbidity or (the need for) adjuvant therapy may preclude this option. All appropriate breast reconstruction options should be offered and discussed with patients, irrespective of whether they are all available locally” [3]. From as early as the 1990s, scientists have emphasized the development of breast reconstruction procedures and techniques together with a growing understanding of the psychological consequences of amputation. These procedures are gaining a permanent position as part of modern, comprehensive breast cancer treatment [22]. However, a crucial aspect of a patient’s quality of life after the procedure is its physical sphere, which is not sufficiently discussed as of yet. Despite the fact that several authors emphasize in their papers that latissimus dorsi breast reconstruction may lead to upper limb functionality disability [3], the literature lacks research on physiotherapy—either after the procedure or before it—for the purpose of primary prevention. Numerous authors showed in their publications that after a one-side mastectomy, previously symmetrical body structure changes, which may be connected to changes in the parameters characterizing body posture. Breast weight influences the body’s gravity center location and removal of one of them thereby causes changes in body posture (elevated shoulders and one-axis trunk inclination) [23,24]. Incorrect body posture may, in turn, result in other somatic changes, while pain and limited mobility of the upper limb causes alterations of body statics [5,6]. It has also been proven in research conducted by Hojan et al. that utilizing mastectomy in oncological treatment may cause alterations in muscle harmony within chest and, in consequence, of postural muscles which may, in turn, cause faulty posture [25]. Trunk asymmetry may also occur, depending on the amount of time that has passed since the procedure. Conducting research on what abnormalities may occur in women after their mastectomy seems perfectly justified considering how often the patients who underwent surgical removal of breast cancer have to face delay of the reconstruction using various oncoplastic techniques. The results obtained via testing with a DASH questionnaire point to decreased functional ability levels of patients who underwent the delayed LDBR during a period of no longer than a year after the procedure, as compared to the control group results. Yang et al. used DASH similarly to assess physical aspects of patients’ post-LDBR life quality [26]. The test was conducted before the surgery, as well as three, six and twelve months after. The degree of disability after a year stayed at a higher level than assessment before the procedure. It should be emphasized that, despite return of the upper limb to the state prior to the procedure in terms of strength and mobility, DASH results were nonetheless worse. Retrospective research from Garusi et al. showed long-term presence of disability in post-LDBR patients assessed with a DASH questionnaire, although it was primarily a disability of minimal or moderate degree. Additionally, it proved that DASH results were lower for patients engaged in sports activities, especially those using the latissimus dorsi [27]. Research by Huizum et al. also showed the significant difference of DASH results between post-LDBR patients and healthy subjects, which additionally correlated with the upper limb muscle strength decrease [28]. Other authors point out the fact of upper limb muscle strength decrease as well [10,29,30]. A systematic literature review from 2014 includes seven papers using DASH to assess functional ability and life quality after procedures using LDF. The papers focused primarily on LDBR procedure and in every paper, the final number of points for the main part of DASH and the additional module discussed in the paper was lower than 20, indicating slight disability in everyday life and work, while the results of an additional module regarding sport/playing an instrument varied greatly—from 2.9 to 84.3 [15]. In another systematic review from 2016, the extreme differences in DASH results of post-LDBR patients were noted [29]. On the basis of the author’s own research as well as research by other authors, it may be assumed that LDF breast reconstruction procedure causes decrease of functionality and life quality, especially in the time period immediately after the procedure. With the passage of time, these limitations partially disappear, however; they can still remain for a long time after the procedure. Unfortunately, there is no agreement on the duration of this stage in the literature. Results obtained in the author’s own research on the shoulder complex active mobility also point to a significant influence of the LDBR procedure on the range of assessed movements. Limiting of the active range of shoulder flexion, abduction, extension and external rotation was determined. Flexion limitation is also proven by the latissimus dorsi flexibility test results. The above-cited systematic review of Min et al., including 22 papers on post-LDBR patients’ functional results, confirms the results of the authors of this paper, simultaneously pointing to limiting of shoulder active ROM. Such results were presented by 13 of the analyzed papers (discussing 369 cases in total). Nine of the analyzed papers reference specific movement directions. They point to shoulder flexion and abduction as the most limited directions. These reports agree with the results of our research. Significant limiting of flexion and abduction movements may result in alterations in overhead activities, which may lead to upper limb function impairment and decrease in quality of life. On the other hand, certain authors fail to point out the connection between LDBR procedure and limiting of shoulder mobility [10,30,31,32]. The results obtained by us point to decrease of motor control of shoulder complex movements in comparison to the control group and the non-operated limb. The assessment of the static position of the scapula also points to postural asymmetry between the operated and non-operated side. A literature review offers no information on motor control of the post-LDBR patients. The only study evaluating scapular position in a group of patients was research conducted by Eyjolfsdottir et al., which indicated increased scapular retraction on the operated side six months after the procedure, decreasing in a subsequent assessment conducted one year after the procedure [33]. Meanwhile, it is emphasized that incorrect motor control and scapular position have influence on shoulder area dysfunction development [34,35]. Our results may thus suggest increased risk of occurrence of such dysfunctions, as well as of upper limb functionality decrease, shoulder mobility decrease and worse motor and postural control of the shoulder in patients after delayed LDBR reconstruction procedure. The crucial issue is surely adjusting the breast reconstruction method to patient body structure and individual needs. A correct qualification by not only a doctor but also a physiotherapy specialist before the planned reconstruction procedure is just as important. It will not only allow you to achieve satisfactory treatment results but will also improve the quality of life of the patient, which is not without merit. The results presented in this paper confirm the necessity of comprehensive implementation of functional assessment of post-mastectomy patients who may undergo LDBR reconstruction procedure. The assessment would be a basis for planning comprehensive physiotherapy and could prevent abnormalities resulting from the preceding breast amputation due to implementation of appropriately early, correct rehabilitation utilizing primary prevention. This in turn could potentially limit or eliminate negative consequences of the delayed LDBR procedure.

## 5. Conclusions

LDBR procedure may have influence on limiting shoulder active mobility, which was also described in further studies, as well as on shoulder motor and postural control decrease. The evidence in motor control assessment in LDBR individuals is scarce in the literature, thus these findings seem to be novel.

Standard functional assessment (consisting of range of motion evaluation, muscle function assessment, physical aspects of quality of life, etc.) of post-mastectomy patients qualified for the LDBR procedure seems to be necessary, but assessment of motor and postural control should be also included.

These results should be confirmed on a larger group of patients. The influence of impaired upper limb motor control on development of shoulder dysfunctions (i.e., pain syndromes) in post-LDBR individuals should be also determined.

## Figures and Tables

**Table 1:** DASH (The Disabilities of the Arm, Shoulder and Hand) questionnaire results.

Group	The Average DASH Questionnaire Result	Level of Significance (*p*)
Group A	60.7 points (from between 30 to 98 points)	*p* = 0.0014
Group B	32.6 points (from between 30 to only 42 points)	

**Table 2:** Scapular position static assessment.

Group	The Ratio of the Distance Between the Scapular Lower Angle and the Spine Line	Level of Significance (*p*)	The Ratio of the Distance Between the Scapular Upper Angle and the Spine Line	Level of Significance (*p*)
Group A	average value of asymmetry was 1.2 cm (from 0.5 to 2.5 cm)	*p* = 0.0010	average value of asymmetry was 1.0 cm	*p* = 0.0134
Group B	average value of asymmetry was 0.45 cm (from 0.3 to 1.2 cm)		average value of asymmetry was 0.45 cm	

**Table 3:** Shoulder active mobility assessment.

Direct of Movement	Group A the Average Range of Motion (ROM) Operated Side	Group A the Average Range of Motion (ROM) Non-Operated Side	Group A the Average Value of the Case Asymmetry	Group B the Average Range of Motion (ROM) for Both Limbs	Group B the Average Value of the Case Asymmetry	Level of Significance (*p*)
Flexion	148.3° (from between 120° to 175°)	164° (from between 153° to 175°)	15.7° (from between 4° to 33°)	166.4° (from between 150° to 174°)	3.8° (from between 1° to 7°)	*p* = 0.0022
Abduction	136.8° (from between 80° to 178°)	172.1° (from 160° to 180°)	35.3° (from 2° to 91°)	168.1° (from between 155° to 178°)	4.0° (from between 1° to 14°)	*p* = 0.0022
Extension	15.9° (from between 7° to 28°)	9.2° (from between 10° to 33°)	3.3° (from between 4° to 8°)	21.3° (from between 17° to 28°	1.8° (from between 1° to 5°)	*p* = 0.0062
External rotation	38° (from between 5° to 75°)	52.1° (from 20° to75°)	14.1° (from between 1° to 29°)	51.75° (from between 25° to 61°)	3.9° (from between 2° to 11°)	*p* = 0.0014

**Table 4:** Latissimus dorsi flexibility assessment.

Group A the Average Range of Motion (ROM) Operated Side	Group A the Average Range of Motion (ROM) Non-Operated Side	Group A the Average Value of the Case Asymmetry	Group B the Average Range of Motion (ROM) for Both Limbs	Group B the Average Value of the Case Asymmetry	Level of Significance (*p*)
146.7° (from between 103° to 168°)	158.27° (from between 136° to 173°)	13.17° (from between 1° to 33°)	164.67° (from between 145° to 174°)	4.0° (from between 1° to 10°)	*p* = 0.0091

**Table 5:** Motor control assessment.

Direct of Movement	Group A the Average Value of the Angle at Which Scapular Control Loss Operated Side	Group A the Average Value of the Angle at Which Scapular Control Loss Non-Operated Side	Group A the Average Value of the Case Asymmetry	Group B the Average Value of the Angle at Which Scapular Control Loss Both Limbs	Group B the Average Value of the Case Asymmetry	Level of Significance (*p*)
Flexion	65.5° (from between 20° to 160°)	108.9° (from between 30° to 175°)	43.4° (from between 10° to 99°)	131.8° (from between 50° to as much as 173°)	16.6° (from between 3° to as much as 115°)	*p* = 0.0036
Abduction	75.8° (from between 20° to 155°)	151.5° (from between 70° to 180°)	75.7° (from between 20° to 139°)	138.75° (from between 50° to 178°)	5.5° (from between 1° to 14°)	*p* = 0.0001
External rotation	28.6° (from 0° to 50°)	50.2° (from between 0° to as much as 75°)	21.6° (from between 1° to 57°)	44.45° (from between 10° to 60°)	2.7° (from between 2° to 20°)	*p* = 0.0006
Extension	12.2° (from between 0° to 20°)	17.2° (from between 10° to 23°)	5° (from between 4° to 10°)	19.2° (from between 5° to 27°)	1.4° (from between 1° to 5°)	*p* = 0.0057
